# Effects of dulaglutide on alcohol consumption during smoking cessation

**DOI:** 10.1172/jci.insight.170419

**Published:** 2023-11-22

**Authors:** Leila Probst, Sophie Monnerat, Deborah R. Vogt, Sophia Lengsfeld, Thilo Burkard, Andrea Meienberg, Cemile Bathelt, Mirjam Christ-Crain, Bettina Winzeler

**Affiliations:** 1 Department of Endocrinology, Diabetology and Metabolism, University Hospital Basel, Basel, Switzerland.; 2 Department of Clinical Research, University of Basel and University Hospital Basel, Basel, Switzerland.; 3 Department of Cardiology, and; 4 Medical Outpatient Department, University Hospital Basel, Basel, Switzerland.

**Keywords:** Endocrinology, Addiction, Neuroendocrine regulation

## Abstract

**BACKGROUND:**

Alcohol use disorder has a detrimental impact on global health and new treatment targets are needed. Preclinical studies show attenuating effects of glucagon-like peptide-1 (GLP-1) agonists on addiction-related behaviors in rodents and nonhuman primates. Some trials have shown an effect of GLP-1 agonism on reward processes in humans; however, results from clinical studies remain inconclusive.

**METHODS:**

This is a predefined secondary analysis of a double-blind, randomized, placebo-controlled trial evaluating the GLP-1 agonist dulaglutide as a therapy for smoking cessation. The main objective was to assess differences in alcohol consumption after 12 weeks of treatment with dulaglutide compared to placebo. The effect of dulaglutide on alcohol consumption was analyzed using a multivariable generalized linear model.

**RESULTS:**

In the primary analysis, participants out of the cohort (*n* = 255) who reported drinking alcohol at baseline and who completed 12 weeks of treatment (*n* = 151; placebo *n* = 75, dulaglutide *n* = 76) were included. The median age was 42 (IQR 33–53) with 61% (*n* = 92) females. At week 12, participants receiving dulaglutide drank 29% less (relative effect = 0.71, 95% CI 0.52–0.97, *P* = 0.04) than participants receiving placebo. Changes in alcohol consumption were not correlated with smoking status at week 12.

**CONCLUSION:**

These results provide evidence that dulaglutide reduces alcohol intake in humans and contribute to the growing body of literature promoting the use of GLP-1 agonists in treatment of substance use disorders.

**TRIAL REGISTRATION:**

ClinicalTrials.gov NCT03204396.

**FUNDING:**

Swiss National Foundation, Gottfried Julia Bangerter-Rhyner Foundation, Goldschmidt-Jacobson Foundation, Hemmi Foundation, University of Basel, University Hospital Basel, Swiss Academy of Medical Science.

## Graphical abstract

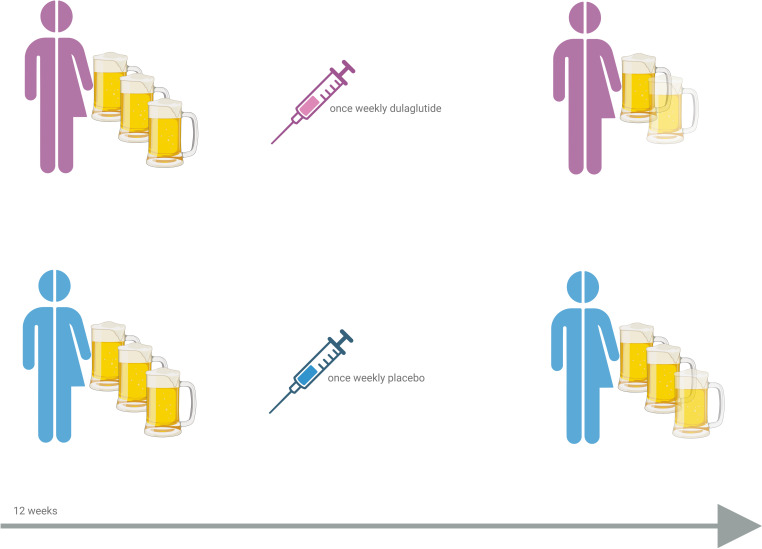

## Introduction

Alcohol use disorder (AUD) affects approximately 5% of people worldwide and is considered a key risk factor for noncommunicable disease, such as a variety of cancers, neuropsychiatric disorders, and liver cirrhosis (1). In 2020, the estimated prevalence of substance use disorders (SUDs), i.e., drug use, other than consumption of alcohol, which leads to dependence and/or requires treatment, was at 0.76% among the population aged 15–64, with an increasing trend between 2016 and 2019. However, when taking into account the past 15 years, SUD prevalence remained relatively stable (2).

There are currently 4 approved pharmacological treatments for AUD, and though these substances display favorable effects in some studies (3), they do not seem to work effectively for all individuals affected by AUD. Considering the complex nature of the disease and the heterogeneity of affected patients (4), it becomes evident that researchers should focus on the identification of new concepts and targets in treating AUD and SUD.

Glucagon-like peptide-1 (GLP-1) agonists have been used in the treatment of type 2 diabetes and obesity due to their incretin and anorexigenic effects. The fact that they reduce appetite has led researchers to investigate their effect on brain circuits that regulate the motivational properties of drugs of abuse. GLP-1–producing neurons in the CNS (5) project to sites of the mesolimbic reward system, which express GLP-1 receptors (6). In 2013, Egecioglu et al. found that the GLP-1 agonist Exendin-4 reduces alcohol intake and alcohol-seeking behavior in rodents and that it attenuated the effect of alcohol on the mesolimbic system (7). These results were largely reproduced using liraglutide (8), dulaglutide (9), and semaglutide (10). Similarly, GLP-1 agonists (exenatide and liraglutide) reduced alcohol consumption in nonhuman primates (11). Furthermore, GLP-1 agonism has been found to attenuate reward induced by other substances, e.g., psychostimulants (12–14) and nicotine (15, 16).

The sole randomized trial in patients with AUD found no group difference regarding reduction in heavy drinking days, but functional MRI (fMRI) cue reactivity and dopamine transporter availability were significantly decreased in the exenatide group compared with placebo (17). Another research group identified a GLP-1 receptor gene variant associated with higher occurrences of AUD and pathological drinking patterns (18). To date, clinical trials have not shown a reduction in cocaine use under GLP-1 agonists, but interestingly, acute cocaine administration was found to reduce GLP-1 serum levels (19, 20). Taken together, these studies suggest that GLP-1 does play an important role in reward-related processes in humans and should be further investigated.

We previously conducted a randomized placebo-controlled trial that investigated whether the GLP-1 agonist dulaglutide increased abstinence rates from nicotine during smoking cessation (21). Smoking abstinence rates at the end of the trial intervention did not differ significantly between the treatment groups. However, whether dulaglutide had an effect on alcohol consumption in this population was unknown. The current study is a predefined secondary analysis aimed to explore whether dulaglutide affects alcohol intake in patients treated for smoking cessation. We hypothesized that 12-week treatment with dulaglutide would lead to a reduction in weekly alcohol intake. This would contribute to the body of evidence promoting the use of GLP-1 agonists in treatment of addictive disorders.

## Results

### Baseline participant characteristics.

Of the full set of 255 participants, 151 (59.2%) consumed alcohol at baseline. Baseline characteristics of the full set and according to alcohol consumption are shown in Supplemental Table 1; supplemental material available online with this article; https://doi.org/10.1172/jci.insight.170419DS1 Of the participants consuming alcohol at baseline (Set 1), 75 were randomized to placebo and 76 to dulaglutide. This subset was composed of 60.9% females (*n* = 92), with a median age of 42 (IQR 33–53) years. Baseline patient characteristics were comparable between groups. Participants in both groups had a median of 20 (placebo: IQR 11.5–35.0; dulaglutide: IQR 11.0–34.0) pack years. Median cigarettes smoked per day in the placebo and dulaglutide groups were 20 (IQR 15–24) and 17.4 (IQR 14–20), respectively. The median Fagerstrom score was 7 (IQR 6–8) in both groups, indicating a high cigarette dependence (22, 23). In both treatment groups, the median alcohol consumption at baseline was 3 (IQR 2–7) standard glasses per week, 67.5% (*n* = 102) of the participants drank wine, 58.3% (*n* = 88) drank beer, and 17.2% (*n* = 26) consumed spirits. One-third (38.4%, *n* = 58) reported drinking more than one type of alcohol. Twelve percent (*n* = 18) were classified as heavy drinkers, i.e., females who drink more than 7 and males who drink more than 14 glasses of alcohol per week (24). Detailed information on baseline participant characteristics from Set 1 is shown in Table 1.

### Alcohol consumption.

**Figure 1:**
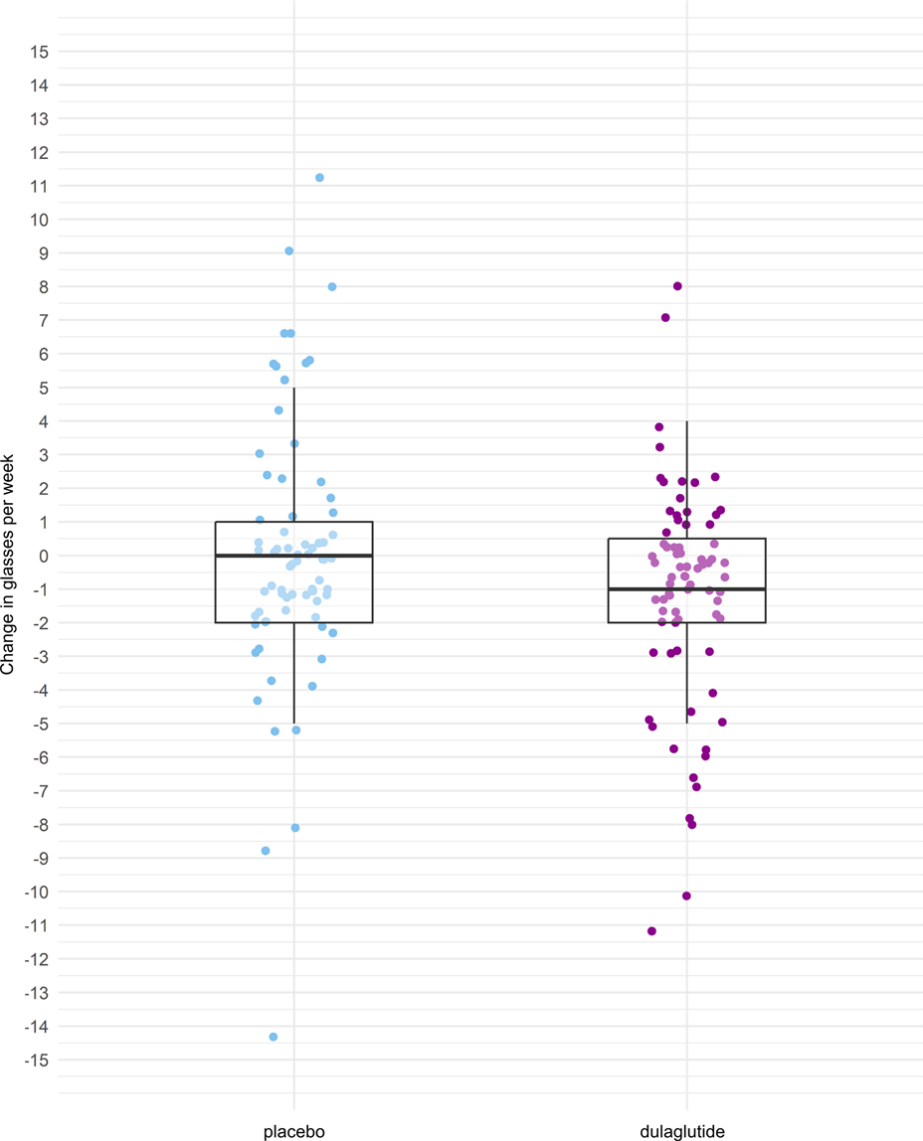
Changes in weekly alcohol consumption. Changes in glasses of alcohol consumed per week from baseline to week 12 according to treatment group. The thick lines represent medians (placebo 0, dulaglutide –1); box indicates the IQR (placebo –2 to 1, dulaglutide –2.25 to 0.25). Whiskers include all data points within the range of 1.5 times the IQR. Note that the figure shows descriptive summary statistics of the data and that dots represent individual data points. Outliers have been removed for better visualization (placebo: +32, –15, –23; dulaglutide: –16).

**Figure 2:**
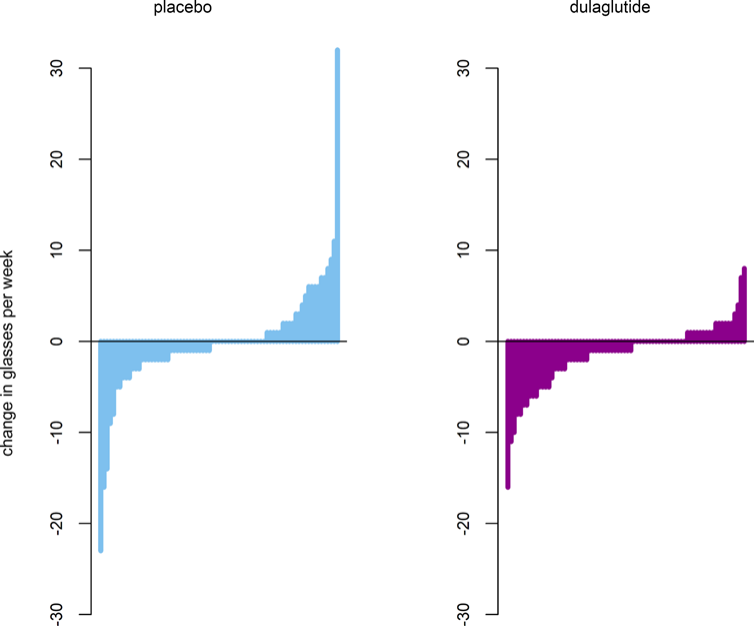
Changes in weekly alcohol consumption. Changes in glasses of alcohol consumed per week from baseline to week 12 for each participant according to treatment group. The bars represent individual data points.

The mean change in weekly alcohol consumption after 12 weeks was –1.4 (SD 3.7) glasses in the dulaglutide group and –0.1 (SD 6.3) glasses in the placebo group. Summary statistics on changes in weekly alcohol consumption according to treatment group are shown in Figure 1. Changes in weekly alcohol consumption from baseline to week 12 and according to treatment group are shown for each participant in Figure 2. At week 12, participants in the dulaglutide group drank an estimated 29% less (baseline alcohol intake adjusted relative effect = 0.71, 95% CI 0.52–0.97, *P* = 0.04) than participants in the placebo group. There was no interaction between baseline alcohol consumption and treatment (*P* = 0.2), meaning that the effect of dulaglutide on alcohol consumption at week 12 did not depend on baseline alcohol consumption. When additionally adjusted for level of education, as suggested by selection via quasi-Akaike Information Criterion (qAIC), the relative effect size of dulaglutide increased to 0.64 (95% CI 0.47–0.86, *P* = 0.004), meaning that patients in the dulaglutide group drank 36% less at week 12 than participants in the placebo group.

### Subgroup analysis of heavy drinkers.

In the subgroup of heavy drinkers (10 in the placebo group, 8 in the dulaglutide group), no group difference regarding changes in alcohol consumption was observed (*P* = 0.5).

### Alcohol consumption and smoking cessation.

**Figure 3:**
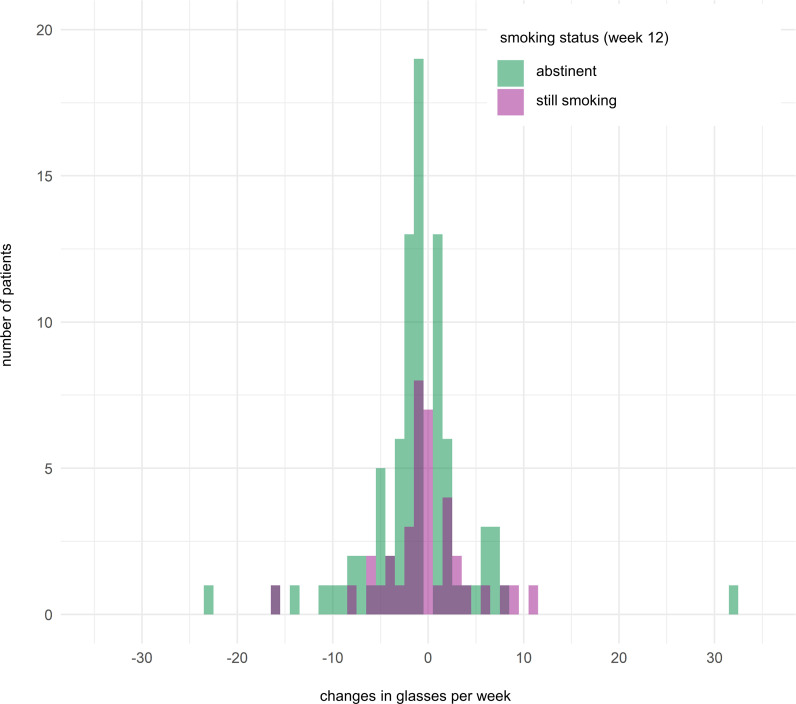
Changes in alcohol consumption according to smoking status. Changes in glasses of alcohol consumed per week from baseline to week 12 according to smoking status at week 12. Each bar represents the number of patients drinking a certain number of glasses alcohol per week.

Changes in weekly alcohol consumption from baseline to week 12 and smoking status at week 12 were not correlated (correlation coefficient 0.045, 95% CI –0.116 to 0.203, *P* = 0.58; Figure 3).

**Figure 4:**
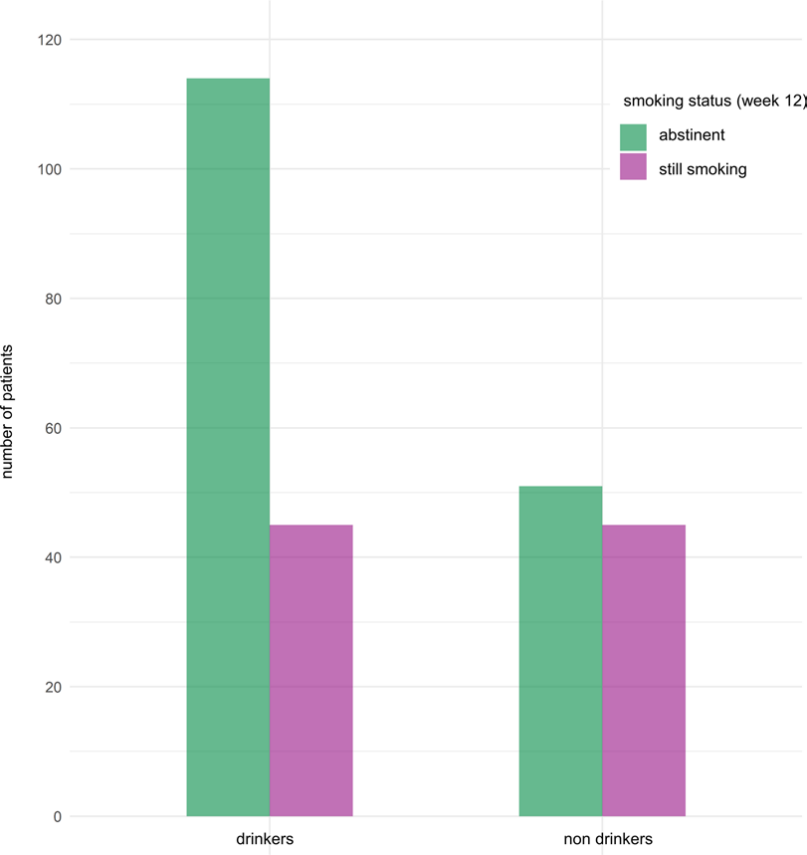
Smoking abstinence rates. Smoking abstinence rates at week 12 according to baseline alcohol consumption. The bars represent absolute numbers of patients.

Abstinence rates from cigarettes at week 12 among alcohol consumers were higher compared with nondrinkers (*n* = 114/159 [72%] vs. 51/96 [53%]; difference in proportions: –0.19 [95% CI –0.32 to –0.06]; *P* = 0.004) (Figure 4).

### New alcohol drinkers.

From the baseline nondrinkers (*n* = 96), 13 participants started consuming alcohol during the study, of which 7 had been randomized to placebo and 6 to dulaglutide. One-third (30.8%, *n* = 4) of this subset was female. The smoking abstinence rate at week 12 was 53.8% (*n* = 7).

### Drug consumption.

At baseline, 10 participants in the placebo group and 12 participants in the dulaglutide group reported consuming drugs (i.e., drugs such as cannabis, benzodiazepines, opioids, psychostimulants, or others), of which 4 in the placebo and 2 in the dulaglutide group quit. Four participants started consuming drugs in each treatment group during the study. There was no evidence to support the hypothesis that the intake of GLP-1 agonists leads to changes in the consumption of drugs (*P* = 0.6).

## Discussion

The main finding of the present study is that participants treated for smoking cessation drink significantly less alcohol after 12 weeks of treatment with dulaglutide compared with placebo, without correlation to smoking status. Our results thus support the hypothesized effect of GLP-1 agonists on alcohol intake in humans.

The role of GLP-1 in reward-related processes has been demonstrated in preclinical studies. GLP-1 receptors are present in the mesolimbic reward system (6) and their activation attenuates alcohol-induced reward and reduces alcohol intake in animals (7, 8, 11, 25). Translating these findings into humans has proven difficult. Treatment with exenatide for 26 weeks compared to placebo did not lead to a greater reduction in heavy drinking days in a randomized controlled trial of patients with AUD. It did, however, decrease fMRI cue reactivity and central dopamine transporter availability (17); hence, there is evidence that GLP-1 agonism affects the human reward system. A potent placebo response, which is often the case in trials for addiction treatment (26), might have reduced the effect size in the above-mentioned study (17) as well as in our main trial (21). The strong placebo response in the study by Kruse-Klausen et al. may in part be explained by the fact that all participants received standard AUD behavioral treatment sessions every second week throughout the trial (17). Indeed, in the main analysis of the present study, participants in both groups achieved unusually high abstinence rates from cigarettes (63% in the dulaglutide group, 65% in the placebo group), independent of treatment allocation. The fact that we found a significant group difference in alcohol intake at week 12 might be explained by participants’ focus on smoking cessation rather than on reducing their drinking, and participants did not suffer from AUD as a primary disorder. This might also explain why results from animal studies cannot be extrapolated to humans. Animals, unlike participants in clinical trials, have no intrinsic motivation to reduce consumption of alcohol and thus display no placebo effect.

The population of the present study was predominantly obese (BMI > 29.9 kg/m^2^; 90.8% overall, 87.0% in the placebo group, 95.2% in the dulaglutide group). Interestingly, although Kruse-Klausen et al. found no overall group difference regarding heavy drinking days in AUD patients in the above-mentioned randomized controlled trial, subgroup analyses revealed that exenatide significantly reduced heavy drinking days in obese individuals (defined as BMI > 30 kg/m^2^) (17). Furthermore, exenatide inhibited brain responses to food cues in obese individuals, but not in lean individuals in a cross-sectional study (27). GLP-1 agonists might therefore affect the reward system differently in obese patients compared with lean individuals.

Participants in our study received standard smoking cessation therapy, i.e., behavioral counselling and varenicline. Behavioral counselling is a form of psychotherapy implemented in treatment of SUD, including AUD. Varenicline has been found to reduce alcohol intake, especially in smokers, but also independently of smoking status (28). These concomitant therapies might have affected alcohol intake in our population. However, all participants received varenicline and behavioral counselling regardless of treatment allocation, and thus we consider group differences in alcohol consumption to be due to the effect of treatment allocation.

In pursuit of alternative reinforcers, patients quitting smoking may increase their consumption of alcohol (29). Furthermore, alcohol may lessen withdrawal symptoms from nicotine (30); hence, one could hypothesize that participants who try to quit smoking would not only drink more alcohol for reward effects, but also curb withdrawal symptoms. Preclinical data suggest that GLP-1 can attenuate withdrawal, as experiments with GLP-1 agonists reduced withdrawal-induced anxiety in rodents (31, 32). If the same were true in humans, GLP-1 agonists would diminish the urge to consume alcohol when experiencing withdrawal symptoms. In the present study, there was no increase in alcohol consumption in the placebo group. One possible explanation might be that participants in both groups received a concomitant therapy with varenicline, which, as mentioned above, has also been reported to reduce alcohol consumption (28, 33). In type 2 diabetes and obesity, GLP-1 agonists are prescribed due to their effect on appetite and homeostatic feeding, and clinical studies report a reduction in fluid intake (34, 35). We did not assess calorie or fluid intake and thus cannot rule out that these mechanisms had an effect on our results. However, 2 facts lead us to believe that the reduction in alcohol consumption under dulaglutide is not solely due to a reduction in overall energy and fluid intake. First, in rodents, GLP-1 agonists reduce consumption of substances that have no caloric value, i.e., psychostimulants; and second, different preclinical studies have shown a decrease in alcohol intake independent of fluid or calorie intake (8, 25, 36). Furthermore, changes in consumption of alcohol and weight changes were not correlated (see supplemental material).

Gastrointestinal symptoms are common side effects of GLP-1 agonists (37–40) and might have affected alcohol intake. However, as expected, these symptoms were only transient and more common in the dulaglutide group at week 2, but comparable at week 12, when alcohol consumption was assessed (21).

Smoking and drinking are closely associated (41–43). It has been hypothesized that nicotine deprivation leads to a higher intake of alcohol in pursuit of alternative reinforcers (29) and attenuates withdrawal symptoms (44). Accordingly, acute nicotine deprivation has been reported to increase the urge to consume alcohol and actual alcohol intake (30). However, other studies indicate that smoking cessation does not lead to increased alcohol intake (45–47); some data even suggest that reducing nicotine consumption may have beneficial effects on alcohol consumption (48). Our finding that smoking status at week 12 was not correlated with changes in alcohol consumption is in line with the majority of literature. However, in contrast to the studies reviewed, most of our participants were not heavy drinkers or AUD patients. Furthermore, high abstinence rates from nicotine in the placebo group might have masked a correlation. Alcohol is a well-established smoking trigger (49, 50). Heavy and binge drinkers are less likely to quit smoking, especially in the short term (51–54). The influence of moderate alcohol consumption on smoking cessation success remains elusive; some studies found no difference in quit rates among moderate and nondrinkers (41, 53–55). In other trials though, and in accordance with our study, moderate drinkers displayed higher abstinence rates compared with those who did not drink (52, 56). Nondrinkers were found to have fewer smokers in their social network (52). Thus, it has been hypothesized that those who start smoking despite their environment not actively enabling them are those who find smoking more rewarding (51). Indeed, smoking patterns of nondrinkers have been described as “heavy, automatic, and characterized by a sense of loss of control” (52).

In analogy to the effects on alcohol-mediated reward, in preclinical studies, GLP-1 agonism has been found to also attenuate reward induced by other substances, i.e., psychostimulants (12–14, 57), nicotine (15, 16), and to some extent opiates, though data on the latter are contradictory (58–60). An experimental study testing the effects of exenatide on cocaine consumption in 13 non–treatment-seeking individuals suffering from cocaine use disorder failed to show a reduction in cocaine self-administration and found no changes in subjective cocaine-related effects (19). However, cocaine consumption has been found to decrease GLP-1 serum levels in humans, and the authors hypothesized that this mechanism leads to sustained cocaine consumption (19, 20). To elucidate the exact mechanisms, and to investigate whether GLP-1 plays a role in addiction to other drugs of abuse, such as cannabis or opioids, more data are needed. In our analysis we did not find an effect of dulaglutide on the consumption of drugs, probably because the number of participants consuming drugs (*n* = 30) was too small.

The present study has limitations. First, our participants did not per se suffer from AUD or SUD and the subgroup of heavy drinkers was too small to provide valuable results. Whether our findings can be extended to heavy- or binge-drinking smokers or nonsmoking AUD patients, and how smoking status influences the effect of GLP-1 agonists on alcohol intake needs to be investigated. Second, our primary endpoint of alcohol consumption is self-reported and therefore subjective. However, we were not able to assess objective measurements to quantify alcohol intake, such as biomarkers, in the present study. Furthermore, we did not assess drinking patterns, such as percent heavy-drinking days, abstinence, time spent drinking, or the context in which our participants drank. Third, in our study, participants received dulaglutide for only 12 weeks. Preclinical data show that alcohol intake was reduced for 3 weeks after discontinuation of dulaglutide in male but not female rats (9). Whether termination of treatment leads to a rebound effect in humans or whether the effects are sustained, and whether sex plays a role in posttreatment effects will need to be explored in future studies.

In conclusion, our analysis showed an effect of a GLP-1 agonist on alcohol intake in humans and our results thus strengthen the rationale for implementing GLP-1 agonists in pharmacological AUD treatment. However, it has to be considered that AUD is characterized by a wide range of drinking behaviors and prevalent comorbid psychiatric disorders, including substance abuse. Thus, treatment strategies need to be multidimensional and personalized, taking into account psychosocial components. To identify the GLP-1 agonist’s role in the treatment of AUD and SUD, further studies are needed, elucidating which patients would benefit and whether GLP-1 agonists improve long-term abstinence.

## Methods

Further details can be found in Supplemental Methods.

### Trial design and participants

This is a predefined secondary analysis of a single-center, randomized, double-blind, placebo-controlled, parallel-group trial conducted at the University Hospital Basel, Switzerland, from June 2017 to June 2022, investigating the effect of a 12-week treatment with dulaglutide on smoking abstinence when added to standard smoking cessation therapy (i.e., varenicline plus behavioral counselling). A detailed description of the study methodology has been published elsewhere (61). In brief, the study included 255 smokers with at least moderate cigarette dependence (defined by a Fagerstrom score of ≥5 points; ref. 22), aged 18 to 75 years, who were willing to quit smoking and willing to undergo treatment with varenicline. Main exclusion criteria were pregnancy, preexisting treatment with GLP-1 agonists, severe renal insufficiency (defined as estimated glomerular filtration rate <30 mL/min/1.73 m²), and unstable psychiatric conditions.

### Study outcomes

The primary outcome in this secondary analysis was the difference in total consumption of standard glasses of alcohol per week after 12 weeks of treatment with dulaglutide compared to placebo. Secondary outcomes were correlation between changes in consumption of alcohol and smoking status at week 12, differences in smoking abstinence rates at week 12 between baseline alcohol drinkers and nondrinkers, number and characteristics of participants who started consuming alcohol from baseline to week 12, and changes in consumption of other drugs (i.e., cannabis, benzodiazepines, opiates, psychostimulants, and others).

### Randomization, study medication, and standard of care

Participants were 1:1 randomized according to a computer-generated randomization list (randomly selected, varying block sizes; no stratification). Participants received a once-weekly, subcutaneous injection of either dulaglutide or placebo. Dulaglutide was injected at an initial dose of 0.75 mg/0.5 mL in the first week and then increased to 1.5 mg/0.5 mL in the following weeks until the end of treatment. The placebo intervention was a 0.5 mL injection of 0.9% NaCl. The standard of care included behavioral counselling according to individual needs and a 12-week treatment with the nicotinic receptor partial agonist varenicline, which was uptitrated to a daily dose of 2 mg.

### Study procedure and assessments

At baseline, data on demographics, comorbidities, including psychiatric diseases and substance abuse, and consumption of alcohol and nicotine were collected via face-to-face interview and a short physical examination was performed. Data on consumption of alcohol, nicotine, and other drugs were also assessed at week 12. Alcohol consumption was assessed with a standardized questionnaire by asking for the number of standard glasses of each type of alcohol (i.e., wine, beer, and spirits) consumed per week on average, analogous to the timeline followback method (62). Standard glasses in Switzerland are defined as 3 dL of beer, 1 dL of wine, or 0.3 dL of spirits, i.e., drinks containing roughly 10 g of pure ethanol (63). Smoking abstinence at week 12 was defined as self-reported 7-day smoking abstinence and end-expiratory exhaled carbon monoxide measurements of 10 ppm or less. For more details, we refer to the study protocol (61).

### Statistics

#### Analysis sets.

**Figure 5:**
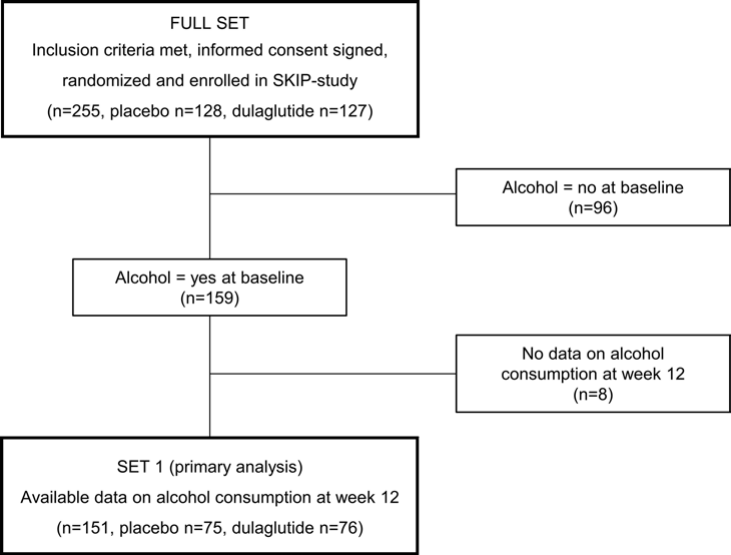
Study flowchart. Study flowchart showing participant selection. The SKIP study is described in Lengsfeld et al. (61).

The full analysis set consisted of 255 participants. According to alcohol consumption at baseline, participants were then divided into alcohol “consumers” and “nonconsumers” (159 and 96 participants, respectively). For the primary outcome, only alcohol consumers with available data on alcohol consumption at week 12 were included (Set 1, *n* = 151; Figure 5).

#### Statistical methods.

Baseline patient characteristics are described using descriptive statistics. Discrete variables are expressed as frequencies (*n*) and percentages (%). Continuous variables are expressed as mean and SD or median and IQR, depending on the data distribution.

The primary outcome, i.e., differences in total amount of alcohol intake in glasses per week at week 12, were analyzed using a generalized linear model with treatment (dulaglutide vs. placebo) and baseline alcohol consumption as predictors (basic model). A quasipoisson error distribution was used to account for overdispersion. The interaction between treatment and baseline alcohol consumption was examined by adding the interaction term to the basic model. The basic model for the primary outcome was further adjusted for education. To assess group differences in changes in alcohol consumption from baseline to week 12 in the subgroup of heavy drinkers, Wilcoxon’s signed-rank test was used. The correlation between changes in consumption of alcohol and smoking status at week 12 was analyzed by calculating the point biserial correlation coefficient. Differences in smoking abstinence rates between baseline consumers and nonconsumers were analyzed using the χ^2^ test. Characteristics of patients who started drinking were summarized using descriptive statistics. Fisher’s exact test was used to test for group differences in drug consumption. For all analyses, a *P* value of less than 0.05 was considered significant. Detailed statistical methods, supplemental analyses, and detailed outputs can be found in the supplemental material. For statistical analyses, the statistics program R v2022.02.1 was used (64).

### Study approval

All participants provided written consent. The study was registered on ClinicalTrials.gov (NCT03204396) and conducted according to the principles of the Declaration of Helsinki. The study protocol was approved by the Ethical Committee Northwest and Central Switzerland (EKNZ 2017-00286) and the national agency for the authorization and supervision of therapeutic products (Swissmedic 2017DR2066).

### Data availability

The data sets used in this analysis are available upon reasonable request. Furthermore, we may share related documents, including the study protocol and the statistical analysis plan. Data will be available with the publication of our manuscript on receipt of a request detailing the study hypothesis and statistical analysis plan. All requests should be sent to the corresponding author. Values for all data points in graphs are reported in the Supporting Data Values file. The steering committee of this study will discuss all requests and decide based on the scientific rigor of the proposal whether data sharing is appropriate. All applicants will be asked to sign a data access agreement.

## Author contributions

LP analyzed and interpreted the data, did the literature search, and wrote the manuscript. SM and LP planned, performed, and interpreted the data analysis. DRV gave important inputs to the statistical analysis plan and supervised the statistical analyses. SM reviewed the manuscript. MCC, TB, and AM were involved in the study design. SL and CB contributed to data collection. MCC gave input to the study design. BW designed the study, wrote the protocol, collected, analyzed and interpreted data, and supervised all steps of the conduct of the study. All authors edited and approved the final manuscript.

## Supplementary Material

Supplemental data

ICMJE disclosure forms

Supporting data values

## Figures and Tables

**Table 1:**
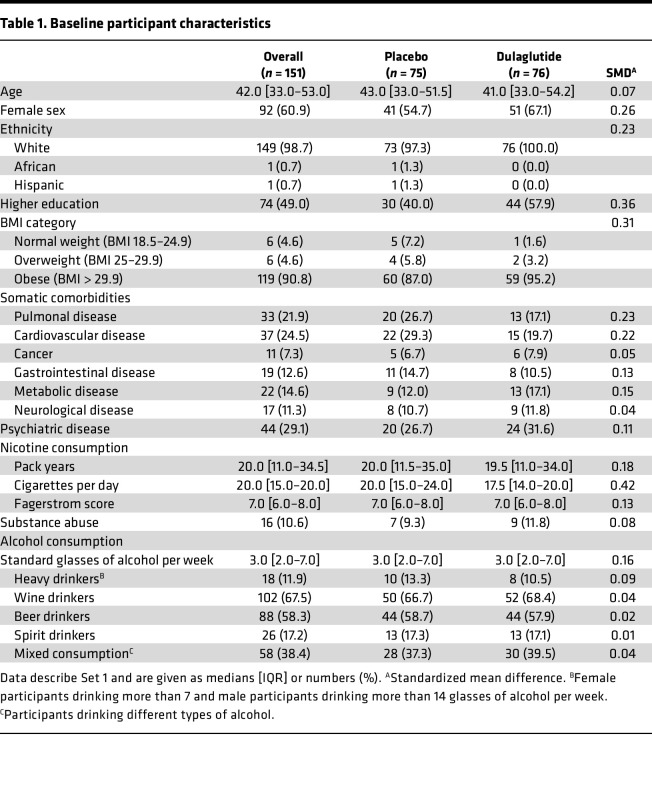
Baseline participant characteristics

## Version 1 11/22/2023

Electronic publication
