# Supplementary material for: Effects of dulaglutide on alcohol consumption during smoking cessation
Source: JCI Insight. 2023 Nov 22;8(22):e170419. doi: 10.1172/jci.insight.170419 (PMC10721313; doi:10.1172/jci.insight.170419)
Supplement: Supplemental data [file jciinsight-8-170419-s182.pdf]

CLINICAL RESEARCH ARTICLE

# Effects of dulaglutide on alcohol consumption during smoking cessation

**Authors:** Leila Probst[1,2], Sophie Monnerat[1,2], Deborah R. Vogt[2], Sophia Lengsfeld[1,2], Thilo Burkard[3,4], Andrea Meienberg[3,4], Cemile Bathelt[1,2], Mirjam Christ-Crain[1,2] and Bettina Winzeler[1,2]

**Affiliations**

[1] Department of Endocrinology, Diabetology and Metabolism, University Hospital Basel, Basel, Switzerland

[2] Department of Clinical Research, University of Basel and University Hospital Basel, Basel, Switzerland

[3] Department of Cardiology, University Hospital Basel, Basel, Switzerland

[4] Medical Outpatient Department, University Hospital Basel, Basel, Switzerland

# Table of Contents

Methods ........ 3
Statistical analysis ........ 3
Supplemental analysis ........ 3
Correlation between weight change and alcohol ........ 3
Model outputs ........ 4
Alcohol consumption ........ 4
Tables ........ 5
Supplementary Table S1: Baseline participant characteristics ........ 5
References ........ 6

# Methods

## Statistical analysis

The primary outcome, differences in total amount of alcohol intake in glasses per week at week 12, was analyzed using a generalized linear model with treatment (dulaglutide vs. placebo) and baseline alcohol consumption as predictors (basic model). A quasipoisson error distribution was used to account for overdispersion. The interaction between treatment and baseline alcohol consumption was examined by adding the interaction term to the basic model. The significance of the interaction term was defined as $p<0.05$. The basic model for the primary outcome was further adjusted for baseline demographics (i.e., sex, age, level of education, baseline drug abuse, baseline BMI category) and the generalized linear model with the lowest quasi Akaike Information Criterion (qAIC) by backward selection using the bbmle package (1) was kept as final adjusted model. Treatment allocation and baseline alcohol consumption were used as fixed predictors, further adjusting for level of education had the lowest qAIC.

To analyze the correlation between changes in consumption of alcohol and smoking status at week 12, the point biserial correlation coefficient was calculated. Patients with missing values on smoking status at week 12 were counted as “still smoking”. Differences in smoking abstinence rates between baseline alcohol consumers vs. non-consumers were analyzed using a Chi-squared test. For this analysis, the full analysis set (n=255) was used. Baseline characteristics and abstinence rates from nicotine at week 12 of patients who started drinking were summarized using descriptive statistics. Fisher’s exact test was used to test for group differences in proportions of participants who either started or stopped consuming drugs, who did not consume at either time point (baseline and week 12) or who consumed at both time points. For this analysis, a different set was used which consisted of all participants with data on drug consumption at baseline and week 12 (n=230).

## Supplemental analysis

### Correlation between weight change and alcohol

The correlation between changes in alcohol consumption and weight was estimated using Spearman’s rank correlation coefficient. Our data suggest that there was no correlation (rho -0.09; p=0.3)

## Model outputs

### Alcohol consumption

**Generalized linear models: Output**

Dependent variable: alcohol consumption at week 12 (glasses per week)

| | basic | adjusted | with interaction |
|---|---|---|---|
| Dulaglutide | 0.71 (0.52, 0.97) | 0.64 (0.47, 0.86) | 0.58 (0.37, 0.89) |
| | p = 0.04 | p = 0.004 | p = 0.02 |
| Baseline alcohol consumption | 1.06 (1.05, 1.07) | 1.06 (1.05, 1.07) | 1.06 (1.04, 1.07) |
| | p = 0.00 | p = 0.00 | p = 0.00 |
| No higher Education | | 0.63 (0.47, 0.84) | |
| | | p = 0.003 | |
| Interaction: treatment:baseline alcohol consumption | | | 1.03 (0.99, 1.07) |
| | | | p = 0.19 |
| Intercept | 3.56 (2.79, 4.48) | 4.52 (3.47, 5.81) | 3.67 (2.87, 4.63) |
| | p = 0.00 | p = 0.00 | p = 0.00 |
| Observations | 151 | 151 | 151 |

# Tables

## Supplementary Table S1: Baseline participant characteristics

Data describe the full set and are given as medians [IQR] or numbers (%).

| | Overall (n=255) | Non-drinkers (n=96) | Drinkers (n=159) |
|---|---|---|---|
| **Age** | 43.0 [33.0, 53.0] | 43.0 [33.0, 53.0] | 43.0 [33.0, 53.5] |
| **Female sex** | 155 (60.8) | 58 (60.4) | 97 (61.0) |
| **Ethnicity** | | | |
| Caucasian | 247 (98.0) | 90 (96.8) | 157 (98.7) |
| African | 2 (0.8) | 1 (1.1) | 1 (0.6) |
| Hispanic | 3 (1.2) | 2 (2.2) | 1 (0.6) |
| **Higher education** | 151 (59.2) | 68 (70.8) | 83 (52.2) |
| **BMI category** | | | |
| normal weight (BMI 18.5-24.9) | 13 (5.7) | 6 (6.5) | 7 (5.1) |
| overweight (BMI 25-29.9) | 12 (5.2) | 6 (6.5) | 6 (4.4) |
| obese (BMI >29.9) | 204 (89.1) | 81 (87.1) | 123 (90.4) |
| **Somatic comorbidities** | | | |
| Pulmonal disease | 57 (22.4) | 20 (20.8) | 37 (23.3) |
| Cardiovascular disease | 70 (27.5) | 30 (31.2) | 40 (25.2) |
| Cancer | 17 (6.7) | 6 (6.2) | 11 (6.9) |
| Gastrointestinal disease | 28 (11.0) | 8 (8.3) | 20 (12.6) |
| Metabolic disease | 48 (18.9) | 24 (25.3) | 24 (15.1) |
| Neurological disease | 25 (9.8) | 8 (8.3) | 17 (10.7) |
| **Psychiatric disease** | 71 (27.8) | 25 (26.0) | 46 (28.9) |
| **Nicotine consumption** | | | |
| Pack years | 20.0 [11.0, 35.0] | 19.5 [10.8, 38.0] | 20.0 [11.0, 35.0] |
| Cigarettes per day | 20.0 [15.0, 20.0] | 20.0 [15.0, 25.0] | 20.0 [15.0, 20.0] |
| Fagerstroem score | 7.0 [6.0, 8.0] | 7.0 [6.0, 8.0] | 7.0 [6.0, 8.0] |
| **Substance abuse** | 24 (9.4) | 7 (7.3) | 17 (10.7) |

## References

1. Bolker B, R Development Core Team. bbmle: Tools for General Maximum Likelihood Estimation. Published 2022. Accessed January 17, 2023. https://github.com/bbolker/bbmle
